# The Hog1 Stress-activated Protein Kinase Targets Nucleoporins to Control mRNA Export upon Stress*

**DOI:** 10.1074/jbc.M112.444042

**Published:** 2013-05-03

**Authors:** Sergi Regot, Eulàlia de Nadal, Susana Rodríguez-Navarro, Alberto González-Novo, Jorge Pérez-Fernandez, Olivier Gadal, Gerhard Seisenbacher, Gustav Ammerer, Francesc Posas

**Affiliations:** From the ‡ Cell Signaling Unit, Departament de Ciències Experimentals i de la Salut, Universitat Pompeu Fabra, E-08003 Barcelona, Spain,; § Centro de Investigación Principe Felipe, Primo Yúfera, E-46012 Valencia, Spain,; ¶ Laboratoire de Biologie Moleculaire Eucaryote du CNRS, Université de Toulouse, 118 route de Narbonne, F-31000 Toulouse, France, and; the ‖ Department for Biochemistry, Max F. Perutz Laboratories, University of Vienna, Dr. Bohrgasse 9, A-1030 Vienna, Austria

**Keywords:** Gene Expression, MAP Kinases (MAPKs), p38 MAPK, Signal Transduction, Stress Response, mRNA Export, Yeast

## Abstract

The control of mRNA biogenesis is exerted at several steps. In response to extracellular stimuli, stress-activated protein kinases (SAPK) modulate gene expression to maximize cell survival. In yeast, the Hog1 SAPK plays a key role in reprogramming the gene expression pattern required for cell survival upon osmostress by acting during transcriptional initiation and elongation. Here, we genetically show that an intact nuclear pore complex is important for cell survival and maximal expression of stress-responsive genes. The Hog1 SAPK associates with nuclear pore complex components and directly phosphorylates the Nup1, Nup2, and Nup60 components of the inner nuclear basket. Mutation of those factors resulted in a deficient export of stress-responsive genes upon stress. Association of Nup1, Nup2, and Nup60 to stress-responsive promoters occurs upon stress depending on Hog1 activity. Accordingly, *STL1* gene territory is maintained at the nuclear periphery upon osmostress in a Hog1-dependent manner. Cells containing non-phosphorylatable mutants in Nup1 or Nup2 display reduced expression of stress-responsive genes. Together, proper mRNA biogenesis of stress-responsive genes requires of the coordinate action of synthesis and export machineries by the Hog1 SAPK.

## Teaser

**Background:** The Hog1 stress-activated protein kinase (SAPK) regulates mRNA biogenesis in response to osmostress.

**Results:** The nuclear pore complex is regulated upon osmostress by the Hog1 SAPK.

**Conclusion:** mRNA export is controlled by the HOG signaling pathway.

**Significance:** Efficient mRNA biogenesis of stress-responsive genes requires of the coordination between synthesis and mRNA export.

## Introduction

In response to stress, cells regulate gene expression to maximize cell survival. Cellular signaling cascades regulate specific transcriptional activities that convert extracellular information into modulation of gene expression (1, 2). Activation of the yeast HOG^8^ (high osmolarity glycerol) signaling pathway in response to osmostress results in a transient and acute regulation of the transcriptional program of the cell (3). The central core of the HOG pathway consists of a tier of kinases highly conserved among eukaryotic cells that results in the phosphorylation and nuclear accumulation of the Hog1 stress-activated protein kinase (SAPK) when extracellular stimuli is sensed (4–6). Transcription profiling studies together with binding analyses of Hog1-dependent transcription factors have shown that Hog1 is a major regulator of the osmostress-regulated transcription that involves a complex and highly specific network of genes (7–15).

The Hog1 SAPK regulates gene expression at multiple levels (3). The SAPK directly phosphorylates several unrelated transcription factors such as Sko1, Hot1, Smp1, or Rtg1/Rtg3 (16–21). Moreover, in response to stress, the kinase associates with several target loci through physical interaction with specific transcription factors (22, 23). Once stably associated with chromatin, Hog1 has diverse functions in the stimulation of gene expression. Recruitment of Hog1 to promoters is essential to coordinate chromatin remodeling and assembly of the transcriptional machinery upon stress (19, 23–26). Moreover, the SAPK physically interacts with elongating RNA polymerase II and cross-links to osmostress-transcribed regions allowing for efficient histone eviction during transcriptional elongation (27–29). In addition, Hog1 has also been involved in the control of mRNA stability as well as in the translation of stress-responsive genes (13, 14, 30, 31).

The nuclear pore complex (NPC) mediates nucleocytoplasmic transport of macromolecules in all eukaryotes (for review, see Refs. 32–34). NPCs have largely symmetrical, doughnut-shape structures that lie within a pore connecting the inner and outer layers of the nuclear envelope. In the yeast *Saccharomyces cerevisiae* the NPC is 50 MDa in size and consists of multiple copies of ∼30 different proteins called nucleoporins (Nups) positioned in a flexible structure (34–37). Not all nucleoporins are equally accessible, and they have completely different roles depending on their location at the scaffold, the nuclear basket, or the cytoplasmic filaments (for review, see Refs. 34 and 38).

Besides trafficking of macromolecules, the nuclear pore has been proposed to exert a direct role in gene expression through physical interactions of Nups with the nuclear genome; indeed, recent work in yeast has revealed that some genes can undergo dynamic recruitment to the periphery upon transcriptional activation (for review, see Refs. 39–42). Localization of individual genes within the nucleus can be dynamically controlled, and many inducible yeast genes such as the *GAL* genes rapidly relocalize from the nucleoplasm to the nuclear periphery after activation by their association with Nups (43–47). Other examples of nuclear pore components interacting with active genes during the initial steps of transcription are described for *INO1* (48–50), *RAP1/GCR1/GCR2* genes (51), or α-factor-induced genes (52). Although many studies pointed out that recruitment to the nuclear periphery appears to have a functional role in promoting transcriptional activation, gene anchoring may not be a general requirement for gene expression (53, 54). For instance, it was reported that repressive environment at the nuclear periphery establishes a negative feedback loop that enables the *GAL* locus to respond rapidly to changes in environmental conditions (55).

In this study we show that nuclear pore integrity is essential for maximal gene expression and adaptation to osmostress. The Hog1 SAPK interacts with and phosphorylates specific components of the NPC to facilitate mRNA export upon osmostress. Therefore, the Hog1 SAPK regulates several steps in mRNA biogenesis from initiation to mRNA export, which might define a dedicated path for optimal expression of osmostress-responsive genes.

## EXPERIMENTAL PROCEDURES

### 

#### 

##### Yeast Strains

*S. cerevisiae* strain BY4741 (MATa *his3-*Δ*1 leu2-*Δ*0 met15-*Δ*0 ura3-*Δ*0*) and its derivatives (*nup42*, *nup100*, *nup53*, *nup157*, *seh1*, *nup188*, *nup170*, *nup133*, *nup120*, *nup84*, *nup2*, *nup60*, *pom34*, *pom152*, *nup59*, *nup1*, and *gle2*) as well as TAP-tagged (Nup42, Nup100, Nup53, Nup157, Seh1, Nup188, Nup170, Nup133, Nup120, Nup84, Nup2, Nup60, Pom34, Pom152, Nup59, Nup1, Gle2, Nic96, Nup116, Nup49, Nup159, Nup57, Nup85, Nup192, Nup145, Gle1, and Nup82) and GFP-tagged (Nup1, Nup2, and Nup60) strains were obtained from the EROSCARF collection. For phospho-mass spectrometry, *NUP2-TAP*::*HIS3 HOG1*::*kanMX4* was obtained. For localization studies, strains were *HOT1-GFP*::*HIS3* with *NUP2*::*kanMX4*, *NUP60* ::*kanMX4*, and *NUP188*::*kanMX4* (YAG200, YAG201, and YAG202), *MSN2-GFP*::*HIS3* with *NUP2*::*kanMX4*, *NUP60*::*kanMX4*, and *NUP188*::*kanMX4* (YAG203, YAG204, and YAG205) and *SKO1-GFP*::*HIS3* with *NUP2*::*kanMX4*, *NUP60* ::*kanMX4*, and *NUP188*::*kanMX4* (YAG206, YAG207, and YAG208). Tagging strains obtained in this study were YSR43 (*NUP1-6HA*::*HIS3*), YSR44 (*NUP1-6HA*::*HIS3 HOG1*::*kanMX4*), YSR218 (*NUP60-6HA*::*HIS3*), YSR221 (*NUP60-HA*::*HIS3 HOG1*::*kanMX4*), YSR38 (*NUP2-6HA*::*HIS3*), YSR47 (*NUP2-6HA*::*HIS3 HOG1* ::*kanMX4*), and YAG209 (*CSE2-6HA*::*KanMX4*). Tagging of genomic ORFs with HA epitope was done with a PCR-based strategy. Strains used for FACS cytometry analysis were YSR237 (*NUP2*::*kanMX4 STL1-qVenus*::*LEU2* pRS413), YSR238 (*NUP2*::*kanMX4 STL1-qVenus*::*LEU2* pRS413-Nup2), YSR239 (*NUP2*::*kanMX4 STL1-qVenus*::*LEU2* pRS413-Nup2^T361A^), YSR325 (*NUP1*::*kanMX4 STL1-qVenus*::*LEU2* pRS413), YSR240 (*NUP1*::*kanMX4 STL1-qVenus*::*LEU2* pRS413-Nup1), and YSR273 (*NUP1*::*kanMX4 STL1-qVenus*::*LEU2* pRS413-Nup1^S11A/T159A/S161A^).

##### Plasmids

The plasmids used in this study were pGEX4T-Hog1 and pGEX4T-Pbs2^EE^ (PBS2 with S514E and T518E mutations), which are described in Bilsland-Marchesan *et al.* (56). NUP1 (PSR19), NUP1^S11AT159AS161A^ (PSR45), NUP2 (PSR3), NUP2^T361A^ (PSR44), and NUP60 (PSR21) were cloned into pGEX4T. RPN2 was cloned into pGEX6P.1 (pAG36). NUP1 (pSR60), NUP1^S11A/T159A/S161A^ (pSR77), NUP2 (pSR57), and NUP2^T361A^ (pSR66) were cloned into pRS413. *STL1-qVenus* construct is described in Pelet *et al.* (57). pRS416 HOG-GFP is described in Ferrigno *et al.* (6).

##### Northern Blot Analysis

Yeast strains were grown to mid-log phase in rich medium at an absorbance at 660 nm of 0.6–0.9 and then subjected to osmostress (0.4 or 1 m NaCl) for the length of time indicated in Figs. 2 and 3. Total RNA and expression of specific genes were probed using radiolabeled PCR fragments containing the entire ORF of *STL1* (1.7 kbp), *CTT1* (1.7 kbp), *GRE2* (1.0 kbp), and the non-coding exon of *RDN18* (1.8 kbp). Signals were quantified with phosphorimaging (Fujifilm BAS-5000), and autoradiographs were obtained on Kodak Biomax XAR film (Sigma).

##### GST Pulldown Experiments

Cells expression expressing GST or GST-Hog1 and specific TAP-tagged proteins grown in mid-log phase were treated with 0.4 m NaCl for 10 min and then collected by brief centrifugation at 4 °C. Pellets were harvested with glass beads in the FastPrep-24 (Qbiogene) in lysis buffer A (50 mm Tris-HCl, pH 7.5, 150 mm NaCl, 15 mm EDTA, 15 mm EGTA, 2 mm DTT, 0.1% Triton X-100, 1 mm PMSF, 1 mm benzamidine, 2 mg/ml leupeptin, 2 mg/ml pepstatin, 25 mm β-glycerophosphate, 1 mm sodium pyrophosphate, 10 mm sodium fluoride, 100 mm sodium orthovanadate), and lysates were clarified by centrifugation and quantified by the Bradford assay (Bio-Rad). As a control, 25 mg of whole-cell extract was blotted with anti-GST antibody to check the expression levels of the tagged proteins. Alternatively, 2 mg of cleared supernatant was incubated with 50 μl of glutathione-Sepharose beads (GE Healthcare) overnight at 4 °C. Beads were washed extensively with buffer A, resuspended in loading buffer, and resolved by sodium dodecyl sulfate-polyacrylamide gel electrophoresis. PAP antibody (Sigma) was used to detect the TAP-tagged proteins.

##### Kinase Assay

The GST fusion proteins encoding NUPs, Hog1, and Pbs2^EE^ were expressed in *Escherichia coli* DH5α and purified using glutathione-Sepharose beads (GE Healthcare) in STET buffer (10 mm Tris-HCl, pH 8.0, 100 mm NaCl, 1 mm EDTA, pH 8.0, 5% Triton X-100, 2 mm DTT, 1 mm phenylmethylsulfonyl fluoride, 1 mm benzamidine, 2 mg/ml leupeptin, 2 g/ml pepstatin). A 1-μg sample of Hog1 was activated with 0.5 μg of Pbs2^EE^ in the presence of kinase buffer (50 mm Tris-HCl pH 7.5, 10 mm MgCl_2_, 2 mm DTT) and 50 mm ATP. After 20 min at 30 °C, eluted NUP protein was added to the previous mixture together with [γ-^32^P]ATP (0.1mCi/ml) and incubated for 15 min at 30 °C. The reaction was terminated by the addition of SDS-loading buffer. Labeled proteins were resolved by SDS/PAGE, stained, dried, and detected by autoradiography.

##### TAP Purification

27 TAP tagged strains were grown to mid-log exponential phase in 250 ml of YPD. Protein extracts were extracted in buffer A as above and incubated with Prot A-Sepharose beads (Sigma) for 2 h at 4 °C. Beads were washed four times with Buffer A and four times with kinase buffer. Kinase assay was performed with Pbs2^EE^-activated GST-Hog1 as above. The reaction was stopped by the addition of SDS-loading buffer. Labeled proteins were resolved by SDS/PAGE, transferred for Western blotting, and detected by autoradiography.

##### Phospho-mass Spectrometry

NUP2-TAP-tagged wild type and *hog1*Δ strains were grown in 1000 ml of YPD to *A*_660_ 2. Unstressed and stressed (0.4 m NaCl, 5 min) cultures were harvested, and pellets were frozen on dry ice. The pellet was resuspended in 8 ml of lysis buffer supplied with protease and phosphatase inhibitors, and standard glass bead lysis was performed. Lysates were cleared by spinning 45 min at 4750 rpm at 4 °C. 0.5-ml IgG beads were added, and the sample was incubated for 120 min at 4 °C. IgG beads were subsequently washed 3 times in lysis buffer supplied with 0.5 m NaCl followed by 3 washes with 1× Tris/DTT. Purified TAP-tagged NUP2 was eluted with 3.5 m MgCl_2_ (1st elution 0.5 ml, 2nd elution 1 ml, and 3rd elution 0.5 ml). The eluate was immediately diluted by adding 3 ml Tris/DTT and concentrated in a Centricon column to 0.5 ml, rediluted with 3.5 ml Tris/DTT, and concentrated to 0.5 ml. The concentrated eluate was precipitated by adding 2 ml of ice-cold acetone, incubated overnight at −20 °C, and pelleted by 14.000 rpm centrifugation for 10 min. Pellet was air-dried and stored at −80 °C. Phosphopeptide enrichment was performed according to Zhou *et al.* (58). No fractionation step was included. 50–100 μg of purified protein was trypsinized and subjected to phosphopeptide enrichment. Phospho-enriched samples were analyzed in a LTQ-Orbitrap, and results were processed with MaxQuant (59). In the case of double-phosphorylated peptides, only intensities of the respective single phosphorylation events that could be unambiguously mapped were analyzed in this study.

##### In Situ Hybridization of Yeast Cells

Localization of *STL1* transcripts was performed following the detailed protocol from the Singer laboratory (64). 5′Cy3-end-labeled 60-bp oligonucleotides were designed covering the ORF region of *STL1* gene. Wild type, *mex67-5*, *nup2*Δ, and *nup60*Δ cells were grown to *A*_660_ 0.5. To inactivate Mex67, the thermosensitive mutant *mex67-5* was shifted to 37 °C for 20 min. NaCl was added to a final concentration of 0.4 m and incubated for 20 min. After salt treatment, the cultures were fixed with formaldehyde, and each strain was used to detect *STL1* RNA specifically. Pictures were taken using a Leica DM6000B fluorescence microscope with a 63× PL APO objective.

##### Chromatin Immunoprecipitation

Chromatin immunoprecipitation was performed as described previously (25). Yeast cultures were grown to early log phase (*A*_660_ 0.6–1.0) before aliquots of the culture were exposed to osmostress (0.4 or 1.2 m NaCl) for the length of time specified in legends. For cross-linking, yeast cells were treated with 1% formaldehyde for 20 min at room temperature. Antibody used in this study was monoclonal anti-HA 12CA5. Quantitative PCR analysis of stress genes and constitutively expressed genes used the following primers with locations indicated by the distance from the respective ATG initiation codon: *STL1* (−372/−112 and +1000/+1280), *CTT1* (−432/−302 and +736/+836), and TEL (telomeric region on the right arm of chromosome VI). Experiments were done on three independent chromatin preparations, and quantitative PCR analysis was done in real time using an Applied Biosystems 7700 sequence detector. Immunoprecipitation efficiency was calculated in triplicate by dividing the amount of PCR product in the immunoprecipitated sample by that in the TEL sequence control. The binding data are presented as -fold induction with respect to the non-treated condition.

##### Statistical Mapping of STL1 Gene Territory

*In vivo* localization of *STL1* locus was performed as previously described (60). Briefly, a mCherry-Nop1 Nup49GFP TetRGFP strain was used to introduce an array of TetR DNA binding sites downstream of the *STL1* locus by homologous recombination. Agar patches of rich media with or without 0.4 m NaCl were used to image cells in cover slides sealed with VaLaP (1/3 Vaseline®, 1/3 lanoline, 1/3 paraffin). Confocal microscopy was limited to 20 min after mounting and using a confocal microscope with an incubator for live cell experiments (The Cube and The Box; Life Imaging Service) at 30 °C and performed with an Andor Revolution Nipkow-disk confocal system installed on a Olympus IX-81 featuring a Yokogawa CSU22 confocal spinning disk unit and a cooled Andor EMCCD camera (DU 888). The system was controlled using the mode “Revolution FAST” of Andor Revolution IQ software (Andor Technology). Images were acquired using an Olympus 100× objective (Plan APO, 1.4 NA, oil immersion). Single laser lines used for excitation were diode-pumped solid state lasers (DPSSL) exciting GFP fluorescence at 488 nm (50 milliwatts, Coherent) and mCherry fluorescence at 561 nm (50 milliwatts, Cobolt Jive^TM^); a Semrock bi-bandpass emission filter (Em01-R488/568–15) allowed collection of green and red fluorescence. Pixel size was 65 nm. For three-dimensional analysis, Z-stacks of 41 images with a 250-nm Z-step were used. Exposure time was 200 ms. Image analysis was done with MATLAB by aligning nuclei using the axis between nucleus and nucleolus three-dimensional centroids. Between 2000 and 3000 cells were analyzed (wild type control, *n* = 3047 cells; wild type NaCl, *n* = 2762 cells; *hog1*Δ control, *n* = 2882 cell; *hog1*Δ NaCl, *n* = 2195). The statistical analyses (*p* value) presented in the figure correspond to a Kolmogorov-Smirnov test testing for equal distribution.

##### Flow Cytometry

Saturated overnight cultures grown in synthetic medium were diluted and grown for 24 h at log phase (below *A*_660_ 0.4). Cells were stressed by mixing 200 μl of culture with 100 μl of stress solution (SD medium + NaCl). Protein translation was stopped after 45 min by the addition of cycloheximide (0.1 mg/ml). Cells were briefly sonicated, and fluorescence was measured by flow cytometry (FACSCalibur, BD Biosciences).

## RESULTS

### 

#### 

##### Nuclear Pore Integrity Is Essential for Growth and Gene Expression upon Osmostress

**FIGURE 1.**
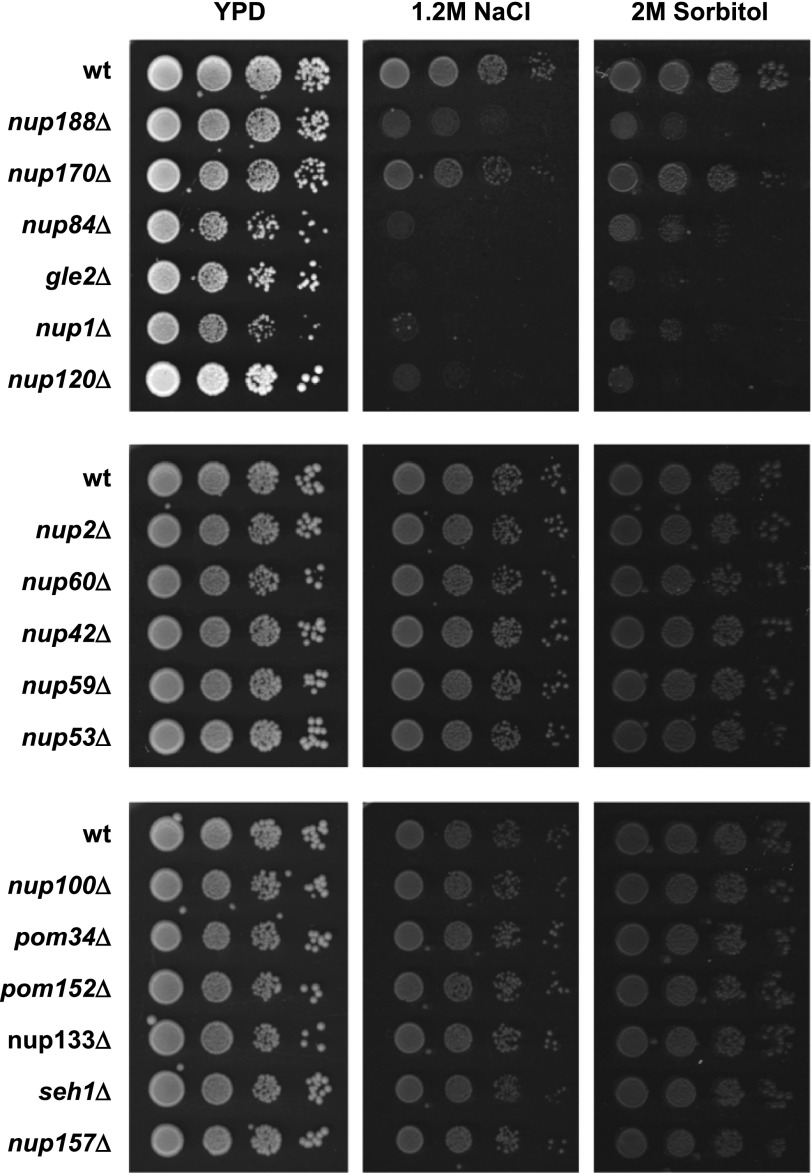
**NPC is essential for growth upon osmostress.** Mutations on specific NPC components render cells osmosensitive. Wild type (*wt*) and the indicated mutant strains were grown to mid-log exponential phase and spotted, making serial dilutions in YPD plates without or with 1.2 m NaCl or 2 m sorbitol. Growth at 30 °C was scored after 3 days.

We performed an exhaustive genome-wide genetic screening searching for mutations that render cells sensitive at high osmolarity to systematically identify novel activities required for full expression of the gene program required for cell survival upon osmostress. This screen identified several transcriptional-related complexes such as the Rpd3 histone deacetylase complex, SAGA, Mediator, the Ubp3 ubiquitin protease, and components of the TEC as well as the RSC complex that are important for gene expression in response to osmostress (24–26, 61). In addition, we also found that mutations in three genes encoding components of the NPC yielded cells osmosensitive (*NUP120*, *NUP170*, and *NUP188*). Then, we systematically extended our screen and monitored cell growth in the presence of high osmolarity in all viable null mutant yeast strains deficient in components of the NPC (17 in total). We assessed the sensitivity of all those strains in 1.2 m NaCl and 2 m sorbitol and found that several mutants in components of the NPC complex (*nup188*Δ, *nup170*Δ, *nup84*Δ, *gle2*Δ, *nup1*Δ, and *nup120*Δ) were sensitive to osmostress (Fig. 1). The mutations included nucleoporins acting as adaptors, coat, cytoplasmic filaments, and components of the nuclear basket.

**FIGURE 2.**
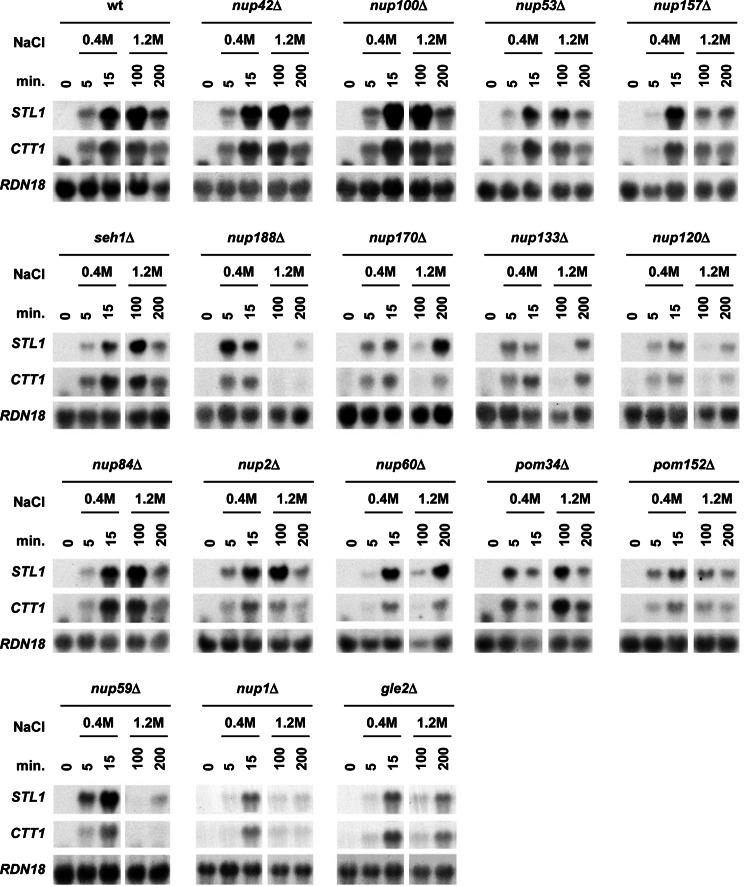
**NPC is essential for gene expression upon osmostress.** WT and the indicated viable NPC mutant strains were grown to mid-log phase in rich medium and subjected to 0.4 m NaCl or 1.2 m NaCl osmostress for the indicated lengths of time. Total RNA was extracted and blotted against *STL1* and *CTT1* and *RDN18* as the loading control.

**FIGURE 3.**
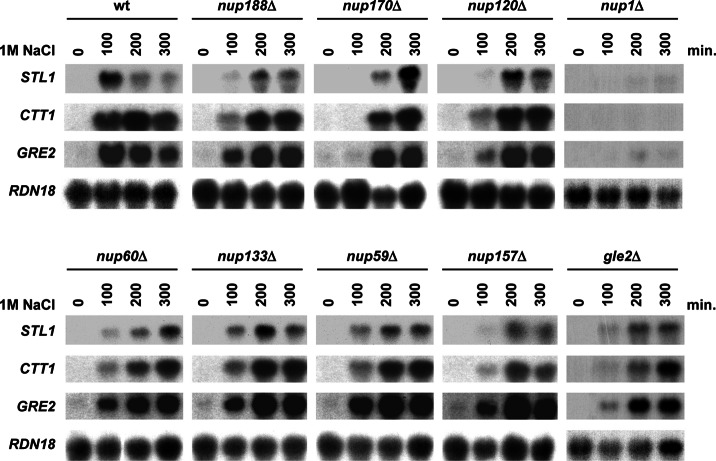
**NPC is essential for gene expression upon osmostress.** WT and the indicated strains were grown to mid-log phase in rich medium and then subjected to osmostress (1 m NaCl) for the indicated length of time. Total RNA was extracted and assayed by Northern blot for transcript levels of *STL1*, *CTT1*, and *GRE2* and *RDN18* as the loading control.

**FIGURE 4.**
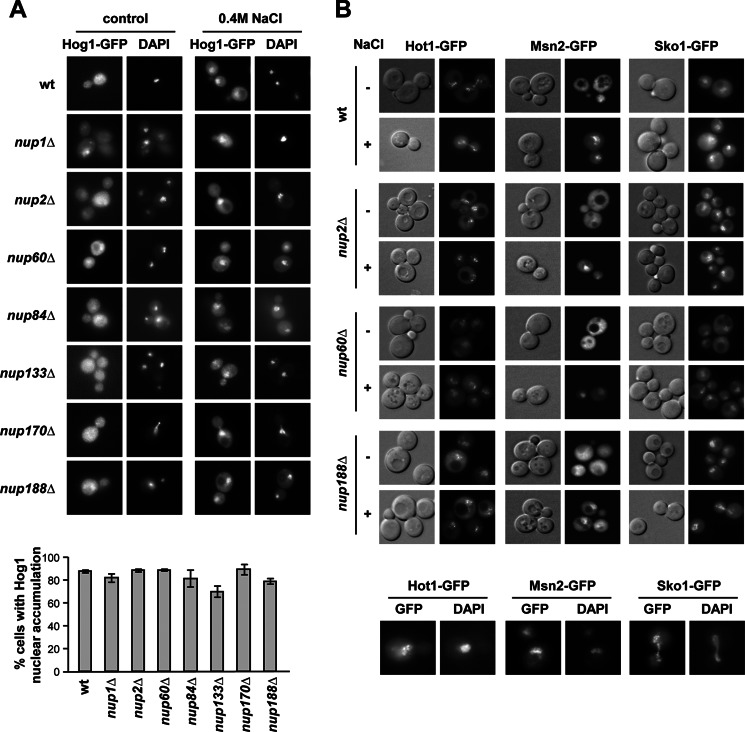
**Localization of Hog1 or transcription factors Hot1, Msn2, and Sko1 is not altered in NPC mutant strains.** *A*, Hog1 localization was not affected in NPC mutant strains. WT and the indicated NPC mutant strains expressing Hog1-GFP were grown in SD medium to mid-log phase and subjected to osmostress (0.4 m NaCl, 5 min). DAPI staining to reveal nuclei and epifluorescence pictures were taken without (basal conditions) and with stress. The percentage of cells with Hog1 nuclear accumulation upon stress is shown in the *lower panel*; data are the mean and S.D. of three independent experiments. *B*, Hot1, Msn2, and Sko1 localization is not affected in NPC mutant strains. WT and the indicated NPC mutant strains expressing Hot1-GFP, Mns2-GFP, and Sko1-GFP were grown in SD medium to mid-log phase and subjected to osmostress (0.4 m NaCl, 5 min). Nomarsky and epifluorescence pictures were taken without (−) and with stress (+). DAPI staining to reveal nuclei in the wild type strain upon stress is shown in the *lower panel*.

We then also assessed systematically whether mutations in components of the NPC resulted in reduced osmostress gene expression. We found that expression of osmoresponsive genes such as *STL1*, *CTT1*, and *GRE2* was significantly affected or delayed under severe osmostress in *nup188*Δ, *nup170*Δ, *gle2*Δ, *nup120*Δ, *nup133*Δ, *nup59*Δ, *nup157*Δ, *nup1*Δ, and *nup60*Δ mutant strains (Figs. 2 and 3). Of note, localization of Hog1 upon stress was not altered in any of the mutant strains tested (Fig. 4*A*). Again, all those mutations included components present in diverse domains of the NPC. The expression of *STL1*, *CTT1*, and *GRE2* is driven by different transcription factors (*i.e.* Hot1, Msn2/Msn4, and Sko1, respectively) and thus indicate a general defect on stress-responsive mRNA accumulation rather than a defect associated with a given transcription factor. Of note, localization of Sko1, Msn2, or Hot1 (fused to GFP) in all nucleoporin mutants analyzed was similar to wild type (Fig. 4*B*). Taken together, our data suggest that a subset of components of the NPC complex, which includes elements in different regions of the NPC, is important for maximal gene expression and cell growth in response to osmostress.

##### The Hog1 SAPK Associates with the NPC Complex

**FIGURE 5.**
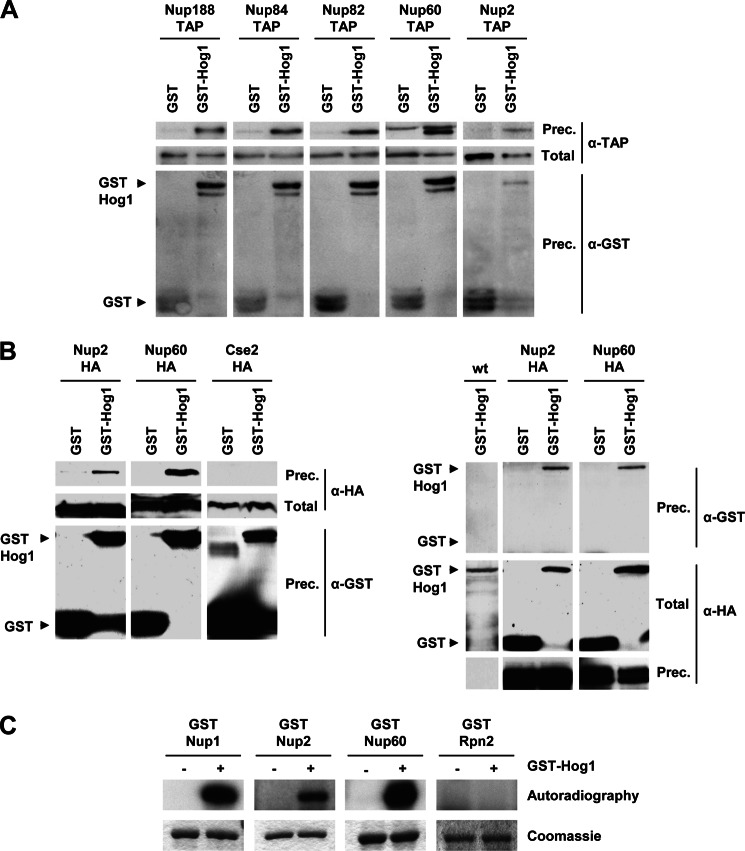
**Hog1 co-precipitates with the NPC and phosphorylates some of its components.** *A*, *in vivo* binding of Hog1 and the NPC is shown. TAP-tagged Nup188, Nup84, Nup82, Nup60, or Nup2 strains expressing GST or GST-Hog1 were grown to mid-log phase and subjected to brief osmotic shock (0.4 m NaCl, 10 min). GST proteins were pulled down by glutathione-Sepharose 4B, and the presence of TAP-tagged proteins was probed by immunoblotting using anti-TAP (PAP; Sigma) (*upper panel*). Total extract represents <20% of total input protein (*middle panel*). Amounts of precipitated (*prec.*) GST proteins were detected using anti-GST (*lower panel*). *B*, GST-Hog1 and Nup2-HA or Nup60-HA reciprocal immunoprecipitations are shown. Nup2-HA and Nup60-HA co-immunoprecipitates with GST-Hog1 in a GST pulldown assay (*left panels*). Cse2-HA was added as negative control. Correspondingly, Hog1 co-immunoprecipitates with Nup2-HA or Nup60-HA in a HA pulldown assay (*right panels*). *C*, *in vitro* phosphorylation of Nup2, Nup60, and Nup1 by Hog1 is shown. Recombinant GST-fused proteins from *E. coli* were phosphorylated *in vitro* by Hog1. A non-relevant protein (GST-tagged Rpn2) was added as a control to exclude possible nonspecific phosphorylation of Nups by Hog1. Phosphorylated proteins were resolved by SDS-PAGE and detected by autoradiography (*upper panel*). Total protein levels were detected by staining with Coomassie Brilliant Blue (*lower panel*).

Hog1 interacts with a number of substrates to control gene expression. The possibility that Hog1 and the NPC complex physically interact was addressed by performing GST pulldown experiments in extracts from osmotically stressed cells expressing GST-Hog1 and a selected group of TAP-tagged nucleoporins (Nup2, Nup188, Nup84, Nup82, and Nup60). In all cases, GST-Hog1 but not the GST control, co-precipitated the TAP-tagged NPC components (Fig. 5*A*). Similar results were obtained when association of GST-Hog1 to HA-tagged Nup2 or Nup60 was assessed in reciprocal co-precipitations (Fig. 5*B*). Thus, the pulldown experiments indicated that Hog1 physically associates with the NPC complex, which provides biochemical evidence for the relationship between NPC and the SAPK.

##### Components in the Nuclear Basket of the NPC Are Phosphorylated by Hog1

Then we used an *in vitro* phosphorylation assay to assess whether Hog1 directly phosphorylates any component of the NPC. Initially, we purified 27 TAP-tag nucleoporins from the yeast TAP-tag collection (Open Biosystems) and subjected them to an *in vitro* kinase assay using *E. coli*-purified Hog1 that was activated in the presence of a constitutive MAPKK allele (Pbs2^EE^) (18). 3 of 27 of the purified TAP-nucleoporins from yeast, Nup1, Nup2, and Nup60 were phosphorylated by the Hog1 SAPK. To validate the results from the TAP assay, we fused Nup1, Nup2, and Nup60 to GST expressed and purified from *E. coli*. All three proteins, but not the Rpn2 negative control, were phosphorylated when they were incubated with active Hog1, indicating that these Nups are direct substrates for the Hog1 SAPK (Fig. 5*C*). Of note, all three target nucleoporins are components of the nuclear basket.

##### Export of Stress-responsive mRNAs Is Altered upon Osmostress in nup2 and nup60 Mutants

**FIGURE 6.**
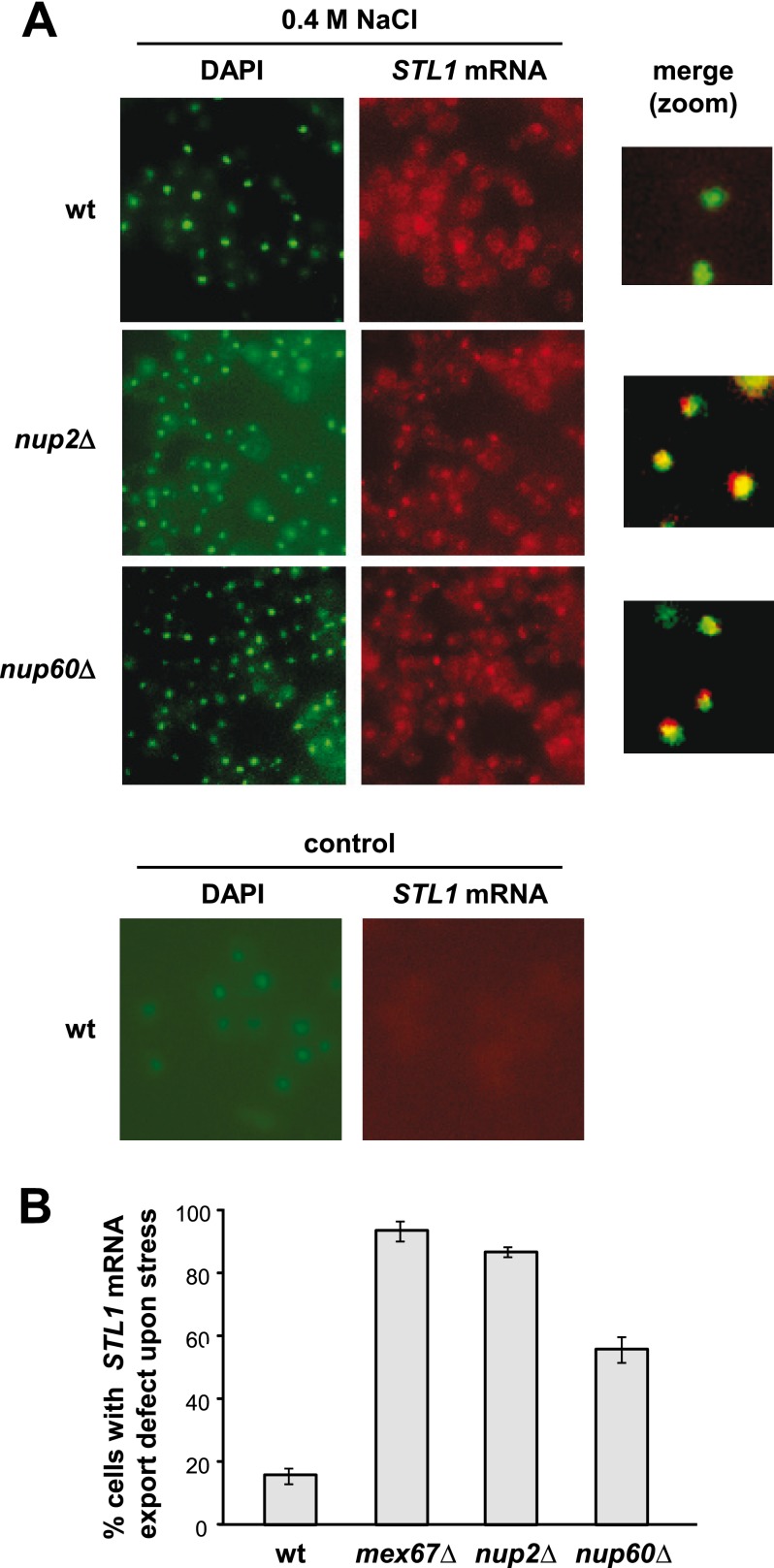
**nup2 and *nup60* mutants display defects in *STL1* mRNA export upon osmostress.** *A*, the localization of *STL1* RNA upon stress (0.4 m NaCl; *upper panels*) and in basal conditions (*lower panel*) was assessed by *in situ* hybridization using Cy3-labbeled probes against *STL1*. DNA was stained with DAPI. A merged zoom image is shown in the *right panels. B*, the percentage of cells with impaired export of *STL1* mRNA in response to osmostress is shown. A temperature-sensitive allele of *MEX67* (*mex67-5*) was used as a positive control of impaired total mRNA export. Data are the mean and S.D. of three independent experiments.

Nucleoporins present in the nuclear basket are important for mRNA export (62, 63). Whereas *nup1* mutant cells show a clear defect in poly(A)^+^ export, mutation of *nup2* and *nup60* only showed a mild poly(A)^+^ export defect in most cells (64). Of note, a subset of cells displayed a more severe defect, suggesting that not all mRNAs were equally dependent on a specific nucleoporin for export (64). To determine whether Nup2 and Nup60 are responsible for stress-responsive mRNA export upon osmostress, *in situ* hybridization of specific *STL1* mRNA was determined. Wild type, *mex67-5*, *nup2*Δ, and *nup60*Δ cells were grown in YPD and subjected to osmostress (0.4 m NaCl) for 20 min. Localization of *STL1* mRNA by *in situ* hybridization of yeast cells was performed using a Cy3-end-labeled *STL1* following standard methods (see “Experimental Procedures”). *STL1* is a highly induced mRNA that is not produced in the absence of stress. As shown in Fig. 6, in contrast to wild type, export of *STL1* mRNA was clearly altered in *nup2* and *nup60* mutant strains in response to osmostress. A temperature-sensitive allele of *MEX67* (*mex67-5*) was used as a control of impaired total mRNA export. Thus, export of the *STL1* stress-responsive mRNA is altered in *nup2* and *nup60* mutants.

##### Nuclear Basket Proteins Nup1, Nup2, and Nup60 Associate with Stress-responsive Genes

**FIGURE 7.**
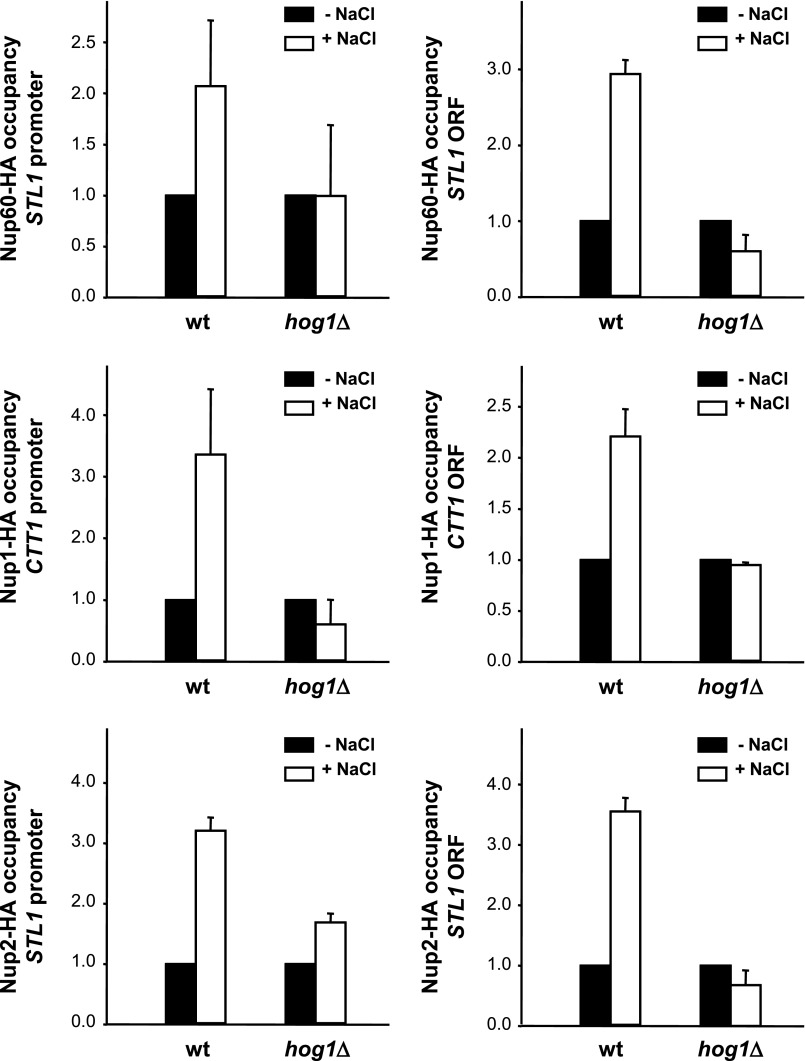
**Nuclear basket proteins bind to stress-dependent genes through Hog1 upon osmostress.** WT and *hog1* strains expressing HA-tagged Nup60, Nup1, and Nup2 were grown to mid-log phase and subjected (*open bars*) to osmostress (0.4 m NaCl, 10 min) or not (*filled bars*). Proteins were immunoprecipitated using anti-HA monoclonal antibody, and binding to the promoter (*left panels*) and ORF (*right panels*) regions of *STL1* and *CTT1* loci was analyzed. The real-time PCR results are shown as -fold induction of treated relative to non-treated (time 0) samples normalized to a telomere internal control. Data are the mean and S.D. of three independent experiments. The statistical significance of the difference was assessed by a paired Student's *t* test of acceptance of equality at (*p* value < 0.05) comparing the Nup binding of a wild type *versus hog1* upon stress.

**FIGURE 8.**
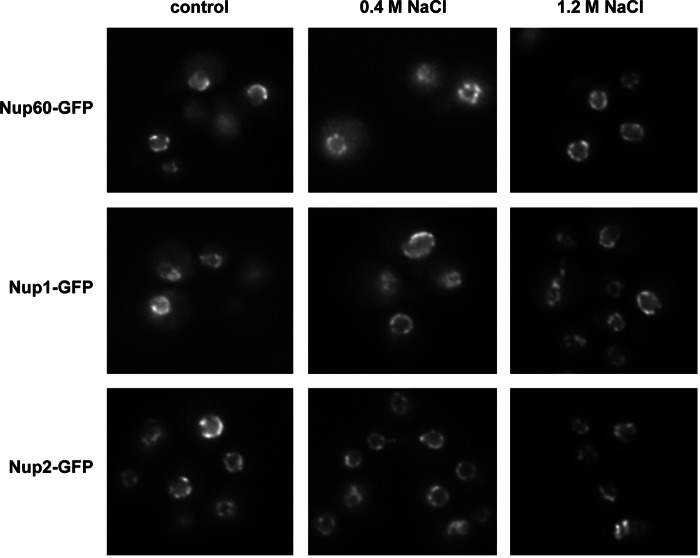
**NPC localization is not affected upon osmostress.** Yeast strains expressing Nup60-GFP, Nup1-GFP, or Nup2-GFP were grown in SD medium to mid-log phase and subjected to osmostress (0.4 m NaCl or 1.2 m NaCl, 5 min). Epifluorescence pictures were taken without (basal conditions) and with stress.

Hog1 associates with stress-responsive gene promoters and throughout the entire transcribed region of target genes in response to stress (22, 28, 29). Because we found that Hog1 interacted with the NPC, we asked whether components of the NPC were also recruited to osmoresponsive genes upon stress. We used chromatin immunoprecipitation to assess the binding of Nup60, Nup1, and Nup2 at promoters and ORFs of osmoresponsive genes (such as *STL1* and *CTT1*) before and after the addition of NaCl. Chromatin from wild type and *hog1* cells, both expressing integrated HA-tagged Nups, was immunoprecipitated and quantified by real-time PCR. As shown in Fig. 7, Nups associated specifically with osmoresponsive promoters and ORFs in response to osmostress. In contrast, no association of Nups was observed in a *hog1* strain, indicating that recruitment of NPC components to chromatin in response to osmostress is dependent on Hog1. Of note, no significant changes in Nup1, Nup2, or Nup60 (fused to GFP) localization were observed upon osmostress (Fig. 8). Taken together, Hog1 is required for NPC association to stress-responsive genes upon osmostress.

##### Hog1 Is Required to Maintain STL1 at the Nuclear Periphery

**FIGURE 9.**
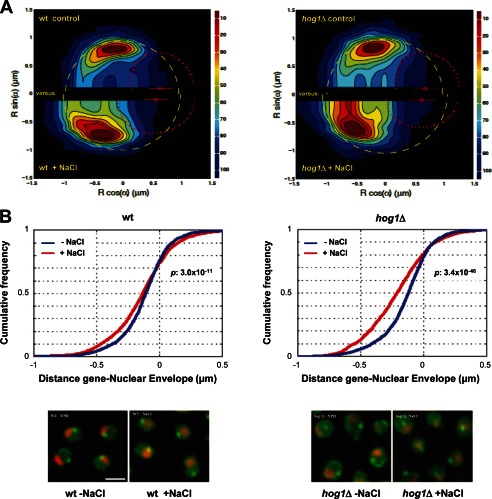
**Hog1 is required to maintain *STL1* at the nuclear periphery.** *A*, shown are *STL1* probability density maps based on analysis of >2,000 nuclei grown in basal (*control*) and 0.4 m NaCl conditions (*upper* and *lower halves of the map*) in WT and *hog1* strains (*right* and *left panels*). *R* indicates STL1 probability density map in cylindrical coordinates. *Dashed yellow circle*, “median” nuclear envelope; *dashed red curve*, “median” nucleolus; *small red circle*, “median” location of nucleolar centroid; *red bar*, range between the percentiles 10 and 90 of the nucleolus centroid position. *Scale bars*, 1 μm. *B*, *STL1* distance relative to the nuclear envelope increases in a Hog1-dependent manner is shown. Radial distance of *STL1* locus to nuclear envelope (cumulative frequencies) is shown in control (*blue*) *versus* 0.4 m NaCl (*red*) conditions in WT and *hog1* strains (*right* and *left panels*). 0 is the nuclear envelop; a negative value indicates a gene position within the nucleus. See “Experimental Procedures” for details. Illustrations of *STL1* localization (*green dot*) images representative of the quantification are shown. *Scale bars*, 2 μm. Shown is peripheral localization of *STL1* compared with nuclear periphery in wt-YPD, wt-NaCl, and *hog1*-YPD and without any preferential nucleolar localization in *hog1*-NaCl.

The association of NUPS at stress-loci prompted us to analyze the location of the *STL1* gene within the nucleus by high resolution statistical mapping using confocal microscopy. This technique permits analysis of the spatial location of a given locus within subnuclear domains (60). We assessed *STL1* location in wild type and *hog1* cells before and after osmostress (0.4 m NaCl for a period of 5–20 min). Live confocal images were gathered and statistically analyzed, and radial distances of *STL1* loci to nuclear envelope were calculated (see “Experimental Procedures”). The *STL1* probability density map (with continuous distribution) based on the analysis is shown in Fig. 9*A. STL1* gene was localized near the nuclear envelope under non-stressed growth conditions in wild type and *hog1* cells, about 20 kb from the Tel4R position (65). In a wild type strain, the distance of *STL1* toward the edge of nucleolus or to the nucleolar center was only affected marginally upon osmostress (the radial distance relative to the nuclear envelope increased slightly; Fig. 9*B*). In contrast, in a *hog1* mutant strain, the relative distance of *STL1* to the nuclear envelope increased markedly. These results would suggest that Hog1 is required to maintain the peripheral location of the *STL1* gene in response to osmostress. Correspondingly, Hog1 is required for the association of Nups to *STL1* loci upon osmostress.

##### Phosphorylation of Nup1 and Nup2 by Hog1 Is Important for Maximal Stress-responsive mRNA Biogenesis

**FIGURE 10.**
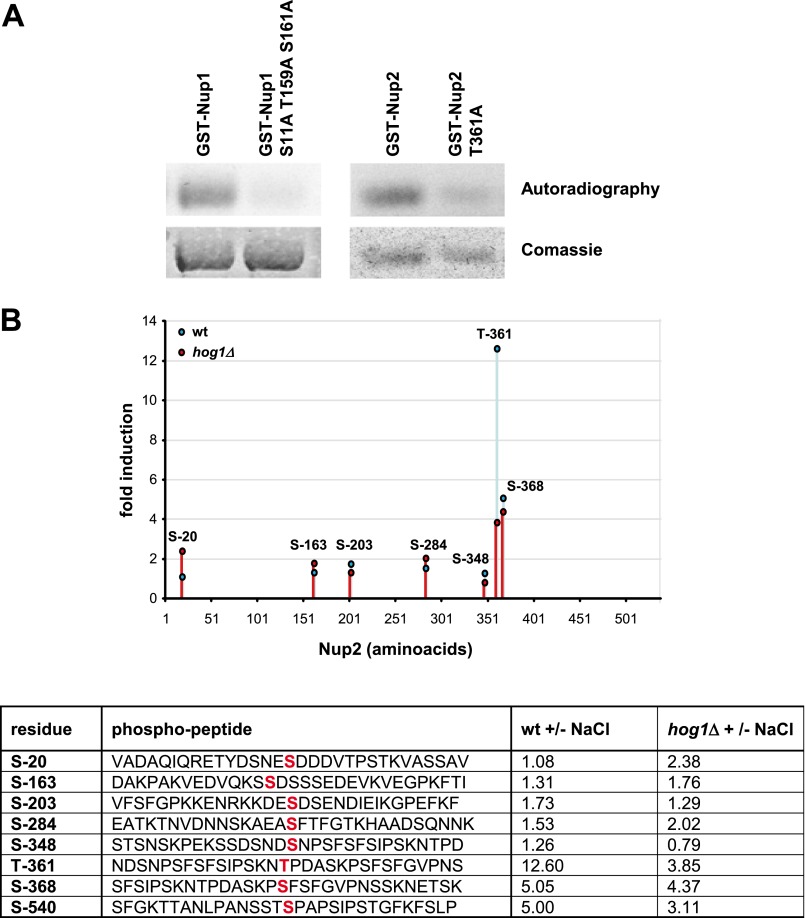
*A*, Nup1 and Nup2 are phosphorylated *in vitro* by Hog1. Recombinant GST-fused wild type and mutant proteins were purified from *E. coli*, and *in vitro* kinase assays were performed as in Fig. 2*B*. Phosphorylated proteins were resolved by SDS-PAGE and detected by autoradiography (*upper panel*). GST-tagged Nup proteins were detected by staining with Coomassie Brilliant Blue (*lower panel*). *B*, shown is -fold change in phosphorylation upon 0.4 m NaCl treatment (5 min) at the indicated residues in Nup2 in wild type (*blue*) and *hog1* mutant (*red*) strains. Ratios have been calculated from absolute phosphorylation intensities of unstressed and osmostressed wild type and *hog1* mutant samples. The table shows the Nup2 residues that have been found to be phosphorylated in this study (*first column*), the position of the phosphorylated residue (*red*) in the MS-detected phosphopeptide (*second column*), and ratios of wild type stressed/wild type unstressed and *hog1* mutant stressed/*hog1* mutant unstressed (*third* and *fourth column*, respectively).

Because several basket nucleoporins were important for mRNA biogenesis and they were also phosphorylated by Hog1, we assessed the relevance of NUP phosphorylation in mRNA biogenesis and export. To identify the phosphorylation sites in the Nup1, Nup2, and Nup60 for the SAPK, several fragments of these proteins were expressed and purified from *E. coli* and then incubated with activate Hog1 in presence of radioactive ATP. Nup1 contained 15 putative Ser-Pro/Trp-Pro sites, whereas Nup2 and Nup60 contained 7 and 9 putative Ser-Pro/Trp-Pro sites, respectively. After systematic mutation analyses we could not significantly reduce the phosphorylation of Nup60 by Hog1 *in vitro*. In contrast, fragment analyses and site-directed mutagenesis of Nup1 and Nup2 permitted the creation of a triple mutant to Ala in Nup1 (*nup1*^S11A/T159A/S161A^) and a single mutant in Nup2 (*nup2*^T361A^) that displayed a strong reduction in the phosphorylation status by Hog1 (Fig. 10*A*). It is worth noting that despite systematic mutagenic analyses of Nup1 and Nup2, we were never able to abolish completely the phosphorylation *in vitro* of these proteins. This suggests that alternative putative phosphorylation sites for Hog1 (either canonical or non canonical) might exist in these proteins.

We then assessed *in vivo* phosphorylation of Nup1 and Nup2 by MS phospho-proteomics. Nup1 was highly problematic for technical reasons, and many of the peptides were not identified in the MS. Nevertheless, we could detect phosphorylation of the Thr-159 and Ser-161 sites *in vivo*. In contrast, Nup2 could be extensively analyzed by MS. Phospho-proteomic analyses showed that Nup2 was phosphorylated at least in eight different sites under non-stress conditions. Remarkably, only phosphorylation of Thr-361 was induced (>10-fold) in response to stress. This phosphorylation was abolished in a *hog1* strain (Fig. 10*B*). Therefore, the Nup2 is phosphorylated at Thr-361 *in vitro* and *in vivo* by Hog1.

**FIGURE 11.**
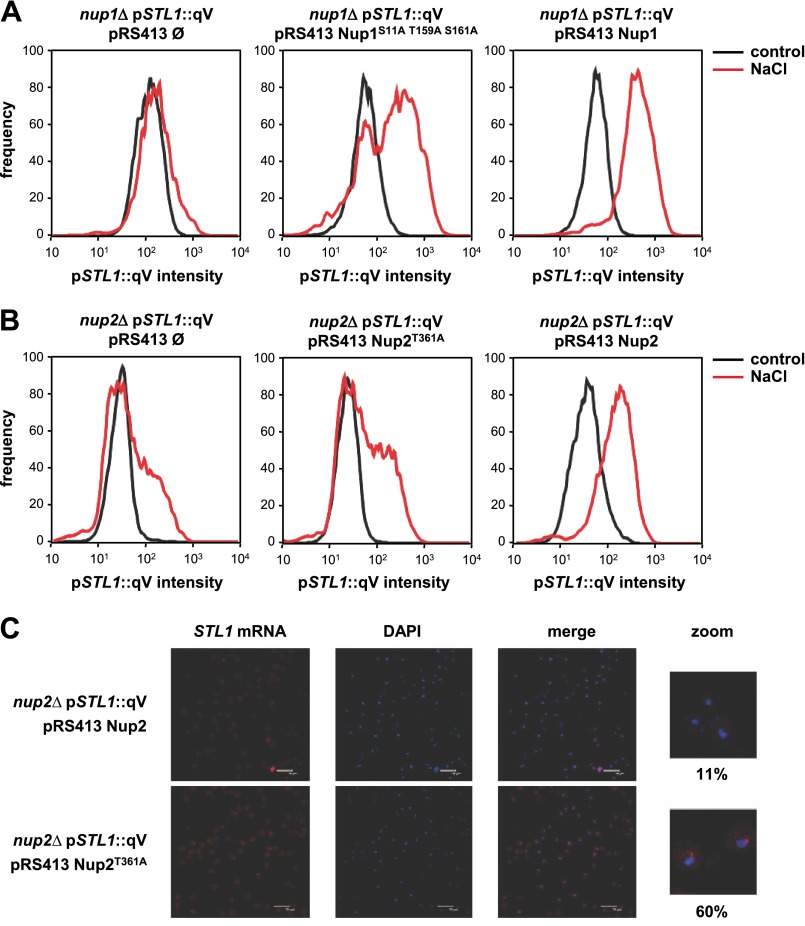
**Phosphorylation of Nup1 and Nup2 by Hog1 mediates proper stress-responsive mRNA biogenesis.** *nup1* (*A*) and *nup2* (*B*) mutant strains harboring the quadruple-Venus (*qV*) fluorescence reporter driven by the *STL1* promoter (p*STL1*) and the indicated low copy number (pRS413) constructs were measured by flow cytometry upon osmostress (1 m NaCl, 70 min). *C*, shown is analysis of nuclear *STL1* mRNA export in the *nup2* mutant strain carrying the *NUP2* wild type or the *nup2*^T361A^ allele upon osmostress. The localization of *STL1* RNA (*left panel*) was assessed by *in situ* hybridization using Cy3-labeled probes against *STL1* in response to 0.4 m NaCl. DNA was stained with DAPI (*middle panel*). Merged images are shown (*right panel*). *Scale bars*, 1 μm (*white line*). Merged zoom images are shown with the percentage of impaired export of *STL1* mRNA in response to osmostress.

Next, we assessed the relevance of the phosphorylation of Nup1 and Nup2 mutations in gene expression. We analyzed gene expression in mutant strains carrying centromeric plasmids carrying the *nup1*^S11A/T159A/S161A^ or *nup2*^T361A^ mutant alleles. We took advantage of a sensitive method to quantify gene expression in single cells. Briefly, we fused the *STL1* promoter to a GFPqv and followed gene expression by flow cytometry (57) in wild type or cells expressing the Nup1^S11AT159AS161A^ and Nup2^T361A^ mutant proteins in the absence or presence of stress. Albeit the maximal degree of expression was similar between the wild type and the Nup1^S11AT159AS161A^ mutant, expression of *STL1* was slower in the mutant when compared with wild type (Fig. 11*A*). In contrast, mutation of the Nup2^T361A^ resulted in both a delay and a reduction in mRNA accumulation that was comparable with the null *nup2* mutant (Fig. 11*B*). Therefore, mutation of the phosphorylation sites by the SAPK in Nup1 and Nup2 resulted in altered mRNA accumulation upon osmostress.

Because the transcriptional defect was more exacerbated in the Nup2^T361A^ mutant, we assessed *STL1* mRNA export by *in situ* hybridization in *nup2*-deficient cells carrying a wild type allele of *NUP2* or the *nup2*^T361A^ mutant. Whereas the wild type strain only displayed 10% of cells with mRNA specks, >60% of the cells carrying the *nup2*^T361A^ mutation presented defects in mRNA export (Fig. 11*C*). Overall, our results suggest that the phosphorylation of nuclear basket nucleoporins by Hog1 is important for proper mRNA export in response to osmostress.

## DISCUSSION

Yeast cells respond to increases in external osmolarity by activating the stress-activated Hog1 SAPK. A major outcome of the activation of Hog1 is the regulation of gene expression, and it has been shown that Hog1 controls gene expression by acting in several steps of mRNA biogenesis (2, 3). Here, we provide evidence that mRNA export, an additional step in mRNA biogenesis, is also controlled by the SAPK.

The identification by genetic means of mutants in the NPC that were osmosensitive and displayed reduced expression of representative stress-responsive mRNAs prompted us to systematically assess which components of the NPC were important for stress adaptation and whether they were targeted by the SAPK. We found that specific mutations in nucleoporins localized in the adaptor, coat, cytoplasmic filaments, and nuclear basket resulted in defects in adaptation and mRNA accumulation upon stress. Mutations in NUPs did not lead to alteration on transcription factor localization. This suggests that integrity of the NPC is important for proper mRNA accumulation upon osmostress. Remarkably, only nuclear basket nucleoporins were directly phosphorylated by the Hog1 SAPK. Thus, this might suggest that several components of the NPC are important for proper stress-responsive mRNA accumulation/export, but only the components that remain at the nuclear side can be directly regulated by the SAPK. Correspondingly, active Hog1 accumulates into the nucleus in response to stress (6).

Nucleoporins have been involved in macromolecular trafficking, proteins, and mRNAs as well as in regulatory functions (33, 66). The phosphorylation of nucleoporins by cyclin-dependent kinases and MAPKs has been reported in yeast and mammals (67, 68). Here, we have shown that the recruitment of nucleoporins occurs at stress loci in response to Hog1 activation. In contrast, Hog1 protein transport is not altered by the deletion of those nucleoporins. Thus, the role of nucleoporin regulation upon osmostress seems to be related to mRNA export. Correspondingly, *STL1* mRNA export is altered upon stress in cells deficient in nuclear basket *nup2* and *nup60* mutants. Furthermore, a point mutation in Nup2 (Nup2^T361A^) that reduces Hog1-dependent phosphorylation also results in defective *STL1* export. Taken together, our data support that mRNA export is controlled by the phosphorylation of NUP components by the SAPK. The fact that null mutations of the NUPs showed a more dramatic effect on gene expression than the non-phosphorylatable mutants could be due to additional phosphorylation sites targeted by the SAPK. Alternatively, it is possible that phosphorylation of the NUPs serves to modulate the efficiency of the transport that would be completely impaired in the null mutants.

Tethering genomic loci to the nuclear envelope has been postulated as a mechanism to optimize gene expression (62, 63, 69). However, the molecular mechanism and functional significance of peripheral localization of transcription-induced genes is poorly understood and whether it is the cause or consequence of transcription activation is still unclear (48, 55, 70). However, several studies have shown a direct link between NPC and gene localization (43, 45–47, 49, 51). Moreover, peripheral localization of certain loci has been linked to NPC phosphorylation. For instance, it has been reported that phosphorylation of Nup1 by Cdk1 alters *INO1* and *GAL1* nuclear localization (68). Here, we have shown that *STL1* is located near the nuclear envelope under normal conditions possibly due to its close proximity to the Ter4R location. However, it is only in response to stress that NUPs associate to the *STL1* loci, indicating the direct tethering of the loci to NPC upon stress. In a *hog1* strain, NUPs do not associate to *STL1*, and the *STL1* locus is not retained at the nuclear periphery. Taken together, our data suggest that association of NPC to stress-responsive loci might serve to optimally couple mRNA production and export. Alternatively, the association of NUPs to promoters might be relevant to stabilize transcriptional complexes at the loci of transcribed genes. Actually, the mutation of the phosphorylation sites for Hog1 in Nup1 and Nup2 seem not only to diminish gene expression but also delays mRNA production. A similar scenario is observed when chromatin remodeling at stress-loci is altered yielding a bimodal response similar to the observed here (57). We cannot formally exclude the presence of nucleoplasmic pool of phosphorylated Nups. In *Drosophila*, the presence of nucleoplasmic Nups away from the NPC, in the nucleoplasm, has been reported (71). Although we cannot directly address this point with *STL1* due to its telomer proximity, it is worth mentioning that upon induction of a telomer proximal gene, *HXK1*, the distance to periphery decreased upon NPC association (46).

In response to stress, general transcription seems to be down-regulated in contrast to an increase of stress-responsive mRNAs (13–15). Thus, the data provided here also point toward the existence of a specialized pathway that would include the control of several steps in mRNA biogenesis. This would favor the production of stress-responsive mRNAs, whereas there is a major down-regulation of mRNA synthesis of general and housekeeping genes. The Hog1 SAPK has been involved in mRNA synthesis, the control of mRNA stability (13, 30), and translation (31). The data provided here suggest that the SAPK also controls the export of stress-responsive mRNAs via the phosphorylation of NUPS at the nuclear basket. Indeed, there are many examples reporting the importance of additional levels in the regulation of gene expression after its synthesis in the nucleus (72). All together, the data support a model by which a single signaling molecule is able to exert the control of several steps in mRNA biogenesis.

## Footnotes

8 The abbreviations used are:

HOG: high osmolarity glycerol
NPC: nuclear pore complex
YPD: yeast extract/peptone/dextrose
qV: quadruple-Venus
TAP: tandem affinity purification.
